# The Effects of Once-Weekly Dulaglutide and Insulin Glargine on Glucose Fluctuation in Poorly Oral-Antidiabetic Controlled Patients with Type 2 Diabetes Mellitus

**DOI:** 10.1155/2019/2682657

**Published:** 2019-12-24

**Authors:** Jie Wang, Hui-qin Li, Xiao-hua Xu, Xiao-cen Kong, Rui Sun, Ting Jing, Lei Ye, Xiao-fei Su, Jian-hua Ma

**Affiliations:** ^1^Department of Endocrinology, Nanjing First Hospital, Nanjing Medical University, Nanjing 210029, China; ^2^National Heart Research Institute Singapore, National Heart Centre Singapore, Singapore

## Abstract

*Aim. *To compare the effects of once-weekly Dulaglutide with once-daily glargine in poorly oral-antidiabetic controlled patients with type 2 diabetes mellitus (T2DM).* Method. *A total of 25 patients with T2DM admitted into Department of Endocrinology from December 2012 to August 2013 were randomly assigned into two groups: Dulaglutide group (*n* = 16) and glargine group (*n* = 9). All patients received either Dulaglutide or glargine treatments for 52 weeks. Continuous glucose monitoring systems (CGMS) were applied to them for two 72 h periods at before and after the treatment each. Patient general clinical data were collected and analyzed.* Result. *Fast blood glucose** (**FBG) of the glargine group declined more significantly than the Dulaglutide group after treatment (*p* < 0.05). The mean blood glucose (MBG), standard deviation of blood glucose (SDBG), mean amplitude of glycemic excursion (MAGE) within a day, the largest amplitude of glycemic excursion (LAGE), *M*-value, absolute means of daily difference (MODD) of glycemic excursion, the percentage of time (≤2.8 mmol/L, ≤3.9 mmol/L, ≥10.0 mmol/L, ≥13.9 mmol/L, 3.9–7.8 mmol/L, and 9–10.0 mmol/L), maximum glycemic value, and minimum glycemic value were similar between the two groups (*p* > 0.05). The incidence of hypoglycemia was also similar between the two groups (*p* > 0.05). Though serum levels of TNF-*α*, IL-6, and 8-PGF2*α* all decreased, significant reduction was found in TNF-*α* and 8-PGF2*α*. TNF-*α* was only significantly reduced in the Dulaglutide group, while 8-PGF2*α* was seen in both groups.* Conclusion. *For T2DM patients with poorly controlled oral antidiabetic drugs, once-weekly Dulaglutide not only has the same effect on glucose fluctuation as once-daily glargine but also significantly reduced TNF-*α* and 8-PGF2*α* after a 52 week treatment protocol. This trial is registered with ClinicalTrials.gov NCT01648582.

## 1. Introduction

According to the International Diabetes Federation (IDF), it is estimated that the number of adult diabetes mellitus in China had reached 109 million, while the number of adult diabetes mellitus in the whole world had reached 415 million [1]. Most of them suffered from type 2 diabetes mellitus (T2DM), which is characterized by insulin resistance, hyperglycemia, and inadequate insulin secretion which results from progressive pancreatic *β*-cell function decline [2]. Currently, there are various drugs used to treat diabetes mellitus, including insulin secretagogues, insulin sensitizer, *α*-glucosidase inhibitor, insulin, or insulin analogues, dipeptidyl peptidase-4 (DPP-4) inhibitor, glucagon-like peptide-1 (GLP-1) analogues, and other oral antidiabetic drugs [3].

Recently, GLP-1 and its analogues have received more attention [4]. GLP-1 is a kind of incretin hormone secreted from L-cells of the intestine when blood glucose increases. Its receptor agonists, such as Exenatide, Liraglutide, Albiglutide, Lixisenatide, and Dulaglutide, activate corresponding signal transduction pathways by combining with GLP-1 receptors on the surface of cells [5]. Different from former GLP-1 receptor agonists which were usually injected 1-2 times a day, Dulaglutide is a new type of GLP-1 receptor agonist which can be injected subcutaneously once a week [6]. Dulaglutide activates GLP-1 receptors, improves glucose-dependent insulin secretion, lowers patient's fasting blood glucose, suppresses the postprandial secretion of glucagon, reduces postprandial glucose, prolongs gastric emptying time, decreases appetite, and improves pancreatic *β*-cell functioning [7].

Recently, glucose fluctuation has been a new indicator in evaluating blood glucose [8]. By activating poly-metabolic pathways, glucose fluctuations can lead to microvascular and macrovascular complications [8]. Currently, few researches have been done on effects of GLP-1 receptor agonists 1–2 times per day on glucose fluctuations, and fewer have been done on effects of once-weekly drugs on glucose fluctuations.

Thus, the present study aims to determine the effect of Dulaglutide subcutaneous injection once a week with that of glargine injection once a day on glucose fluctuations of T2DM patients who were treated unsatisfactorily with metformin and/or sulfonylureas (SU) using the continuous glucose monitoring system (CGMS).

## 2. Methods

### 2.1. Subjects

This study was conducted at Department of Endocrinology; Nanjing Hospital affiliated to Nanjing Medical University, Nanjing. It was approved by the ethical committee of Nanjing Hospital. Written informed consent was obtained from all patients. Twenty-five patients with T2DM admitted into the Department of Endocrinology during the period between Dec. 2012 and Aug. 2013 were recruited as subjects of the present study. Patients were randomly assigned into two groups: the Dulaglutide group (*n* = 16) and the glargine group (*n* = 9), and treated for 52 weeks. 72 h CGMS was applied at 3 days before starting and 3 days after completing the Dulaglutide or glargine treatments. The inclusion criteria were: (1) Patients were >18 years old; (2) Patients with diagnosed T2DM for at least 6 months based on the World Health Organization's (WHO) criteria [9]; (3) Patients had been taking metformin and/or sulfonylurea for at least 3 months and have been taking them on a stable therapeutic dose (at least half the maximum dose) for at least 8 weeks; (4) Patients had a HbA1c level between 7.0% and 11.0%; (5) Patients had a Body Mass Index (BMI) level between 19.0 and 35.0 kg/m^2^; (6) Patients had stable body weight in the past 3 months. The data are stored at Department of Endocrinology; Nanjing Hospital affiliated to Nanjing Medical University, Nanjing and is available upon request.

The exclusion criteria were: (1) Patients had either been treated with a GLP-1 receptor agonist or a GLP-1 analogue, or is currently taking DPP-IV inhibitor and thiazolidinedione (TZD), or is currently taking insulin; (2) Patients had severe diabetic gastroparesis or have taken drugs that directly affect gastrointestinal motility for a long time; (3) Patients had one of the following diseases: acute or chronic hepatitis, acute or chronic pancreatitis, abnormal renal function, a serum calcitonin concentration higher than 20 pg/mL; (4) Patients had a history of malignancy.

### 2.2. Study Protocol

#### 2.2.1. Anti-Diabetic Drugs

Sixteen patients were randomized to receive once a week 0.75 mg (11 cases) and 1.5 mg (5 cases) Dulaglutide (Dulaglutide group). Patients and physicians were blinded to the dose of Dulaglutide. Nine patients received daily glargine (Lantus, Germany) (Glargine group). If patients' FBG ≥7.8 mmol/L, the initial dose of glargine was 6 unit/day (U/D). Insulin dose was adjusted once or twice weekly. The glargine algorithm had a treat-to-target strategy [10], based on the average of the previous three FBG values.

#### 2.2.2. Patient General Data

Gender, duration of diabetes mellitus, height, weight, BMI, blood pressure (BP), heart rate, corrected QT interval of electrocardiogram (ECG), and therapeutic schemes were collected by blinded physicians.

#### 2.2.3. CGMS

Before and after 52 weeks of treatment, we applied CGMS (Medtronic MiniMed, blind CGMS) to the two groups for 72 hours to monitor their continuous glucose. CGMS sensor was inserted under the patients' abdominal skin and connected it to a glucose recorder to detect the glucose concentration of the interstitial fluid and have the data recorded. A mean value for each 5 minutes was stored and 288 measured values were recorded automatically every day with the glucose value, which can be effectively detected ranging from 2.2 to 22.2 mmol/L. The procedure was carried out for 72 hours. During the same period, capillary blood glucose levels of finger tips were measured at least 4 times a day to correct data from CGMS. The data were transmitted to a computer through an information collector and analyzed with the CGMS software 3.0. 2. Patients were required to have the same time for diet and the same volumes of excise food intake during the period of CGMS study. The definition of hypoglycemia: blood glucose is lower than 3.9 mmol/L of blood glucose and lasts for 15 minutes. Patients would measure finger peripheral blood glucose at least 4 times/day; record the incidence of hypoglycemia. Patients would be suggested to take food if blood glucose <3.9 mmol/L.

The following parameters were calculated:

(a) 24 hours mean blood glucose (24  hours-MBG) and standard deviation of blood glucose (SDBG): the average level and SD of a total of 288 measured glucose values of 24  h with CGMS.
(b) Mean amplitude of glycemic excursion (MAGE), the largest amplitude of glycemic excursion (LAGE), *M* value and Absolute Means of Daily Difference (MODD).
(c) Percentage time of hypoglycemia and hyperglycemia (≤2.8 mmol/L, ≤3.9 mmol/L, ≥10.0 mmol/L, ≥13.9 mmol/L, 3.9–7.8 mmol/L, 3.9–10.0 mmol/L).
(d) The maximum blood glucose(MAX-BG) and the minimum blood glucose (Min-BG).

#### 2.2.4. Laboratory Examination

Fasting insulin (Ins 0) and fasting C-peptide (C-P0) were examined by chemiluminescence (Roche-E170, Roche, USA). HOMA-IR was calculated as Ins0 (*μ*IU/mL) × Glu0 (mmol/L)/22.5. HbA1c was examined by High Performance Liquid Chromatography (HPLC: VARIANT2 Automatic hemoglobin tester, Biorad, USA). Index of biochemistry was examined by full automatic biochemical analyzer (Kodak 750, USA). Blood routine examinations were measured with automatic blood cell analyzer (Mindray DC-5380, China). Tumor necrosis factor-*α* (TNF-*α*) was measured using the humanspecifc Milliplex map kit (Millipore, St. Charles, MO, USA). Interleukin-6 (IL-6) was determined using commercially available Enzyme-Linked Immunosorbent Assay kits according to the manufacturer's instructions (R&D Systems, Minneapolis, MN, USA). 8-iso prostaglandin F2*α* (8-iso PGF2*α*) was measured using an enzyme immunoassay method (Cayman Chemical Co., Ann Arbor, MI).

### 2.3. Statistical Analysis

All statistical analyses were done using the SPSS statistical program, version 22. Continuous variables were examined by *t*-test and were presented as mean ± standard deviation (SD). Nonnormal distributed data received the Mann-Whitney test and were presented with interquartile range (IQR). *χ*^2^ test was used to compare the categorical variables, and the statistical results were represented as *χ*^2^-values and *p*-values, and *P* < 0.05 was considered significant.

## 3. Results

### 3.1. Patient General Data

There are no statistical differences found in history of diabetes mellitus (years), sex, age, body weight, BMI, BP, heart rate, ECG Corrected QT interval (*P* > 0.05) between the two groups (Table 1) on week 0. No differences in body weight, BMI, BP, heart rate, and ECG Corrected QT interval (*P* > 0.05) on the 52^nd^ week were found (Table 1). After 52 weeks, changes of BMI were 0.09 (–0.69, 0.53) kg/m^2^ in the Dulaglutide group and 0.73 (0.27, 1.37) kg/m^2^ in the glargine group, respectively, and no statistically significant difference was found (*P* = 0.055). Other microvascular complications, other comorbidities and other treatments were similar between patients of two groups (Table 5).

### 3.2. Laboratory Test

There are no statistically significant differences in FBG, HbA1c, Ins-0, C-P0, HOMA-IR, amylase, lipase, pancreatic enzymes, liver and renal function, blood lipid, blood routine (white blood cell, platelet) between the two groups at baseline. However, statistically significant differences were only in hemoglobin, hematocrit and red blood cell counting. No statistical differences were found between the two groups for any measured parameters after 52 weeks.

By comparing variations of data on the 53^rd^ week with those at baseline levels, we found significant differences in FBG, total amylase, and lipase between the two groups. The reduction of FBG of the glargine group 3.7 mmol/L was significantly bigger than that of the Dulaglutide group's (0.8 mmol/L, *p* < 0.05). The reduction of HbA1c was similar in the two groups: the Dulaglutide reduced from 8.65 ± 1.5% to 7.16 ± 1.17%, while the glargine group reduced form 8.73 ± 0.64% to 7.40 ± 1.12%. The concentrations of total amylase and lipase of the Dulaglutide group rose more significantly than those of the glargine group. The concentration of total amylase of the Dulaglutide group increased from 69.44 ± 26.80 unit/L (U/L) to 80.44 ± 29.99 U/L, while that of the glargine group decreased from 64.78 ± 17.35 U/L to 59.44 ± 13.71 U/L. The concentration of lipase of the Dulaglutide group increased from 43.94 ± 11.60 U/L to 50.50 (38.50, 59.00) U/L, while that of the glargine group declined from 41.44 ± 14.15 U/L to 37.00 (24.00, 55.50) U/L (Table 2).

### 3.3. CGMS Data

There are no statistically significant differences found in MBG, SDBG, LAGE, MAGE, *M*-Value, MODD, and PT (≤2.8 mmol/L, ≤3.9 mmol/L, 3.9–7.8 mmol/L, 3.9–10.0 mmol/L, ≥10.0 mmol/L, ≥13.9 mmol/L). Max-BG or Min-BG either on baseline or after 52 weeks were similar (*P* > 0.05). The two groups' variations in 24 h-MBG from baseline levels were −2.95 (−4.23, −1.93) for the Dulaglutide group and −3.50 (−4.85, 0.55) for the glargine group, respectively, with no statistically significant differences. Variations from baseline levels of the two groups in MAGE were −1.79 (−3.68, 2.74) for the Dulaglutide group and 0.32 (−2.88, 3.21) for the glargine group, but no statistically significant differences were found (Table 3).

### 3.4. Oxidative Stress and Inflammatory Profile

To determine the effect of different drug treatments on oxidative stress and inflammation, we measured TNF-*α*, IL-6, and 8-PGF2*α*. Patients in all groups had higher inflammatory cytokine levels at baseline. After 52 week treatment, TNF-*α* levels significantly decreased in the Dulaglutide group (*p* < 0.05) (Table 4); however, this was not seen in the glargine group. Though there were trends of IL-6 to reduce, no significant difference was found in both groups at 52 weeks compared with those at baseline. Serum 8-PGF2*α* levels significantly decreased in both the Dulaglutide and glargine groups after 52 week treatment as compared with baseline (*p* < 0.05) (Table 4). There were no significant differences found between groups at 52 weeks after treatment.

## 4. Discussion

The present study showed that that addition of once-weekly Dulaglutide has the same effect as once-daily glargine on control of glucose fluctuations and oxidative stress and inflammation in patients with T2DM, who were poorly controlled by oral antihyperglycemic medications.

As a GLP-1 receptor agonist, Dulaglutide consists of GLP-1(7–37) covalently linked to an Fc fragment of human IgG4, thereby protecting the GLP-1 moiety from inactivation by dipeptidyl peptidase 4. The average biological half-life of Dulaglutide is 90 h, making it an ideal candidate for delivery once a week. Currently, few studies have been done on the effects of Dulaglutide on glucose fluctuation, among which the AWARD-4 trial studied Dulaglutide in 884 patients with T2DM [11]. The patients were randomly assigned to receive a 52 week combinational treatment of insulin lispro with once-weekly Dulaglutide or once-daily glargine with insulin lispro. It showed that Dulaglutide in combination with lispro resulted in a significantly greater improvement in glycemic control than glargine did. A substudy of this patient cohort (*n* = 144) received CGMS on weeks 0, 13, 26, and 52, respectively, to monitor blood glucose fluctuation [12]. It showed that the reduction of 24 hours MBG was similar among the three groups at weeks 26 and 52. Though the percentage of time (PT) of normoglycaemia (3.9–7.8 mmol/L) was similar, the percentage of near-normoglycaemia of the Dulaglutide-1.5 mg group (3.9–10.0 mmol/L) was higher than the glargine group on week 26. However, the PTs of normoglycaemia and near-normoglycaemia were similar on week 52. It suggested that the once-weekly Dulaglutide treatment has similar effects on glucose control to that of the once-daily glargine treatment. The study by Probstifield [13], applied CGMS to 102 T2DM patients with high cardiovascular risk to determine blood fluctuation in patients received basal insulin with twice-daily Exenatide or with bolus insulin after 26 weeks. It was found that the indexes of the two groups had similar MAGE and SD.

Hypoglycemia is the most common adverse events, and severe hypoglycemia was closely correlated to mortality [14, 15]. In the present study, the occurrence of hypoglycemia of the Dulaglutide group was similar to that of the glargine group. This is consistent with AWARD-3 and -5 studies, which did not find difference in occurrence of hypoglycemia in Dulaglutide injection and oral-drug taking groups [16, 17]. Comparing the effects of the short-acting Exenatide with that of the fast-acting insulin on hypoglycemia, Jeffery's study did not find significant differences between the two treatments [13]. Instead, the AWARD-4 sub-study found that the glargine group had a higher rate of nocturnal hypoglycemia on week 26. However, the percentage time of hypoglycemia of the glargine group (PT ≤ 3.9 mmol/L) was significantly higher than the Dulaglutide group on week 52, suggesting that the Dulaglutide group had less incidence of hypoglycemia. The AWARD-4 trial found that on weeks 26 and 52, the total rate of hypoglycemia was similar between groups [11]. Similarly, AWARD-2 trial [18] found that on week 52, the total rate and the total times of hypoglycemia and nocturnal hypoglycemia of the glargine group were all higher than those of Dulaglutide groups. Araki et al. [19] found that the total rate of hypoglycemia and nocturnal hypoglycemia of the glargine group were higher on week 26 in Japanese population. Thus, it seems that Dulaglutide has better control on hypoglycemia as compared with glargine.

Abdul–Ghani et al. [15], randomly assigned 231 poorly glycemic-controlled T2DM patients to receive once a week Exenatide/pioglitazone combinational treatment or basal/bolus insulin group for 18 months. They found the rate of hypoglycemia in patients received basal/bolus insulin injection was threefold of that of the patients in the combinational group. Diamant et al. [22] randomly assigned 586 T2DM patients to once-weekly albiglutide group or thrice-daily prandial insulin lispro group. Compared with that of the insulin group, the weekly combinational therapy group had less incidence of hypoglycemia [20]. The present study showed that the occurrence of hypoglycemia of the Dulaglutide group was similar to that of the glargine group, but was different from the Abdul–Ghani's study [15]. It might be due to the different treatments applied in the present study, as higher risk of hypoglycemia may be associated with basal/bolus insulin injection as compared with oral anti-diabetic drugs. Different population races and diet habits may also contribute to the difference.

The present study found that FBG of the glargine group decreased significantly than that of the Dulaglutide group on week 52. AWARD-2 trial [18] compared the safety and efficacy of Dulaglutide (0.75 mg or 1.5 mg) with that of glargine in combination with metformin and Glimepiride. FBG of the glargine group declined more than the Dulaglutide 0.75 mg group on week 52 on AWARD-2. Similarly, AWARD-4 trial found that the FBG of the glargine group declined more on weeks 26 and 52. FBG in the current study was consistent with them, but was different from Araki study in which the FBG of the Dulaglutide group was similar to that of the glargine group on weeks 14 and 26 [19]. The differences may be due to the difference patient population selected in this study and different treatment protocol.

In the present study, HbA1c of the Dulaglutide group declined to a similar level to that of the glargine group on week 52. This is consistent with others [12, 13]. Araki et al. [19] even found that the HbA1c of the Dulaglutide group declined more than that of the glargine group. This might be due to differences in patient population selection and treatment protocol. The present study found that the Dulaglutide group had a similar rate of HbA1c-control (percentage of HbA1c ≤ 6.5% and HbA1c ≤ 7%) to that of the glargine group on week 52. This is consistent with AWARD-4 study which found no statistical differences in rate of HbA1c-control of the three groups [11]. On the contrary, Abdul-Ghani et al. [15] found that higher rates of HbA1c-control were all seen in the weekly-Exenatide group. The difference might be due to differences in clinical protocol, race and sample size.

Decreased oxidation stress and inflammation markers decreased in both groups after 52 weeks' treatment, notably in Dulaglutide group. This suggests that both treatments have anti-inflammatory and oxidative stress effects in T2DM and Dulaglutide has more effective therapeutic efficacy in reducing inflammation in T2DM.

Body weight reduction has been seen in both Dulaglutide and Exenatide treatments [7, 11, 18, 19, 22], Abdul-Ghani found that the body weight gain of the weekly-Exenatide group was only half of that of the control group, with the difference being of statistical significances in Qatar study [15]. Other studies found body weight loss of the Dulaglutide group [11, 18, 19]. Some studies [20, 22] found, when comparing glucose control of once-weekly-albiglutide with that of glargine and comparing twice-daily Exenatide with that of glargine, and found that patients received Exenatide had significant lower body weight. In the present study; however, no significant differences in body weight reduction was found between the two groups at week 52. This might be due to the small sample-size of the present study.

Studies showed increase in amylase, lipase, and pancreatic enzymes in AWARDs studies and Araki's studies [11, 16–19, 23]. The present study also found that the amylase and lipase of the Dulaglutide group increased significantly as compared with those of the glargine group. However, we did not find statistically significant differences in pancreatic enzymes of the two groups. These were consistent with Araki's studies [19]. No adverse events such as acute pancreatitis, pancreatic carcinoma etc., were found during study course [24]. The mechanism of pancreatic enzymes might be due to the increased replication of pancreatic duct cell with obesity and T2DM [25].

The study has several limitations. Firstly, the sample size is small. Secondly, the mechanism of Dulaglutide on reduction of inflammatory biomarkers is unknown. Future study is needed to explore the underlined mechanism.

In conclusion, for T2DM patients with poorly controlled oral antidiabetic drugs, once-weekly Dulaglutide not only has the same effect on glucose fluctuation as once-daily glargine, but also significantly reduced TNF-*α* and 8-PGF2*α* after a 52-week treatment protocol.

## Figures and Tables

**Table 1:** Clinical characteristics.

		Dulaglutide group (*n* = 16)	Glargine group (*n* = 9)	*t*	*P*
Sex (female%)	Baseline	6 (37.50%)	4 (44.44%)		1
History (years)	Baseline	8 (5, 14.5)	5(3, 11.5)	−1.08	0.293
Age (years)	Baseline	59.88 ± 8.17	65.44 ± 11.09	−1.438	0.164
Height (cm)	Baseline	165.13 ± 7.48	159.44 ± 6.21	1.93	0.066
Weight (kg)	Baseline	68.97 ± 10.13	62.56 ± 5.72	2.023	0.055
	52w	69.06 ± 9.85	64.67 ± 6.20	1.205	0.24
Body mass index (kg/m^2^)	Baseline	25.22 ± 2.67	24.63 ± 2.12	0.57	0.574
	52w	25.26 ± 2.53	25.46 ± 2.30	−0.198	0.845
Systolic BP (mmHg)	Baseline	134.38 ± 13.93	133.67 ± 11.94	0.128	0.899
	52w	134.69 ± 16.91	135.44 ± 10.00	−0.122	0.904
Diastolic BP (mmHg)	Baseline	80.06 ± 8.65	77.44 ± 8.53	0.73	0.473
	52w	82.38 ± 8.98	77.89 ± 8.59	1.217	0.236
Heart rate (bpm)	Baseline	78.56 ± 7.20	73.33 ± 6.75	1.781	0.088
	52w	77.50 ± 6.76	75.44 ± 11.25	0.574	0.571
Corrected QT interval	Baseline	438.52 ± 27.42	431.90 ± 18.22	0.636	0.531
	52w	437.00 ± 26.59	427.44 ± 25.41	0.876	0.39

BP: blood pressure.

**Table 2:** Biochemical characteristics.

			Dulaglutide group (*n* = 16)	Glargine group (*n* = 9)	*t*	*p*
Fasting blood glucose		Baseline	10.26 ± 2.84	10.72 ± 2.34	−0.418	0.68
(mmol/L)		52w	8.21 ± 1.70	6.92 ± 1.60	1.85	0.077
HbA1c (%)		Baseline	8.65 ± 1.57	8.73 ± 0.64	−0.186	0.854
		52w	7.16 ± 1.17	7.40 ± 1.12	−0.507	0.617
Total amylase (U/L)		Baseline	69.44 ± 26.80	64.78 ± 17.35	0.467	0.645
		52w	80.44 ± 29.98	59.44 ± 13.71	1.974	0.061
Pancreatic enzymes		Baseline	29.00 ± 7.91	28.22 ± 9.85	0.216	0.831
(U/L)		52w	30.50 (28.25, 37.50)	24.00 (20.00, 31.00)	−2.212	0.026
Lipase (U/L)		Baseline	43.94 ± 11.60	41.44 ± 14.15	0.477	0.638
		52w	50.50 (38.50, 59.00)	37.00 (24.00, 55.50)	−1.728	0.087
Cholesterol (mmol/L)		Baseline	4.81 ± 1.04	4.67 ± 1.05	0.324	0.749
		52w	4.68 ± 1.10	4.79 ± 1.35	−0.221	0.827
HDL-cholesterol (mmol/L)		Baseline	1.24 ± 0.21	1.31 ± 0.34	−0.654	0.52
		52w	1.21 ± 0.28	1.13 ± 0.34	0.602	0.553
LDL-cholesterol (mmol/L)		Baseline	2.82 ± 0.81	2.54 ± 1.02	0.768	0.45
		52w	2.68 ± 0.87	2.27 ± 0.80	1.168	0.255
Triglyceride (mmol/L)		Baseline	1.64 ± 0.87	1.97 ± 1.63	−0.655	0.519
		52w	1.57 (0.88, 2.45)	1.50 (0.92, 5.866)	−0.142	0.901
Calcitonin (ng/L)		Baseline	2.24 ± 0.85	2.57 ± 0.73	−0.954	0.35
		52w	2.13 ± 0.53	2.11 ± 0.33	0.103	0.919
		Change	0 (0, 0)	−0.30 (−0.70, 0)	−2.074	0.052
White blood count (^∗^10^9^/l)		Baseline	6.36 ± 1.42	5.84 ± 1.37	0.889	0.383
		52w	6.54 ± 1.48	6.19 ± 1.03	0.634	0.532
Haemoglobin (g/L)		Baseline	149.63 ± 11.77	139.89 ± 9.69	2.106	0.046∗
		52w	147.63 ± 13.57	139.33 ± 10.71	1.573	0.129
Haematocrit (g/L)		Baseline	0.45 ± 0.04	0.42 ± 0.03	2.167	0.041^∗^
		52w	0.45 ± 0.04	0.42 ± 0.03	1.706	0.102
Red blood count		Baseline	4.91 ± 0.35	4.46 ± 0.50	2.65	0.014 ^∗^
(^∗^10^12^/l)		52w	4.88 ± 0.39	4.52 ± 0.42	2.16	0.041
Platelet count (^∗^10^9^/L)		Baseline	207.63 ± 68.46	180.11 ± 40.20	1.266	0.218
		52w	212.06 ± 59.10	195.33 ± 30.66	0.931	0.361
Blood urea nitrogen		Baseline	5.51 ± 1.34	5.55 ± 1.17	−0.078	0.938
(mmol/L)		52w	5.42 ± 1.19	5.43 ± 1.68	−0.021	0.984
Creatinine (*μ*mol/L)		Baseline	73.50 ± 15.48	65.56 ± 12.51	1.314	0.202
		52w	73.06 ± 16.34	66.78 ± 14.23	0.965	0.345
Alanine aminotransferase		Baseline	24.25 ± 13.93	17.67 ± 3.35	1.8	0.089
(U/L)		52w	21.00 (15.25, 36.50)	22.00 (14.50, 35.50)	−0.142	0.901
Glutamate aminotransferase		Baseline	20.38 ± 5.84	16.78 ± 4.21	1.62	0.119
(U/L)		52w	21.50 (18.00, 27.00)	23.00 (17.50, 25.50)	−0.199	0.857
Microalbumin		Baseline	22.50 (16.00, 52.25)	30.00 (11.00, 70.50)	−0.085	0.944
(mg/L)		52w	26.00 (9.25, 47.25)	41.00 (9.50, 61.50)	−0.538	0.607
Microalbumin/creatinine		Baseline	2.70 (1.13, 6.85)	3.80 (1.15, 12.45)	−0.566	0.588
(mg/g)		52w	3.30 (0.78, 4.55)	4.60 (1.25, 6.55)	−1.076	0.295
Fasting insulin (nmol/L)		Baseline	8.75 (5.30, 12.68)	7.90 (6.00, 14.00)	−0.113	0.923
Fasting C-peptide (nmol/L)		Baseline	0.52 ± 0.31	0.41 ± 0.20	0.888	0.384
HOMA-IR (%)		Baseline	0.93 ± 0.35	0.96 ± 0.38	−0.185	0.855

HOMA-IR: homeostatic model assessment of insulin resistance.

**Table 3:** CGMS results.

		Dulaglutide group (*n* = 16)	Glargine (*n* = 9)	*t*	*p*
PT2.8	Baseline	0 (0, 0)	0 (0, 0)	0	1
	52w	0 (0, 0)	0 (0, 0.50)	−1.925	0.054
AUC2.8	Baseline	0 (0, 0)	0 (0, 0)	0	1
	52w	0 (0, 0)	0 (0, 0)	−1.333	0.182
PT3.9	Baseline	0 (0, 0)	0 (0, 0)	−0.15	0.88
	52w	0 (0, 0)	0 (0, 8.00)	−0.446	0.656
AUC3.9	Baseline	0 (0, 0)	0 (0, 0)	0	1
	52w	0 (0, 0)	0 (0, 0.05)	−1.925	0.054
PT3.9–7.8	Baseline	6.00 (0, 28.50)	10.00 (1.00, 33.00)	−0.605	0.545
	52w	51.50 (27.00, 62.75)	44.00 (22.50, 66.50)	−0.482	0.63
PT3.9–10	Baseline	35.50 (4.25, 49.75)	36.00 (15.50, 53.50)	−0.34	0.734
	52w	80.50 (66.00, 91.75)	56.00 (38.50, 86.50)	−1.359	0.174
PT10	Baseline	64.50 (49.50, 95.75)	64.00 (46.50, 84.50)	−0.34	0.734
	52w	19.50 (8.00, 34.00)	25.00 (11.50, 54.50)	−1.048	0.295
AUC10	Baseline	2.05 (1.23, 4.50)	1.80 (1.05, 2.75)	−0.425	0.671
	52w	0.30 (0.10, 1.28)	0.40 (0.10, 2.45)	−0.913	0.361
PT13.9	Baseline	25.00 (6.00, 41.25)	18.00 (8.00, 36.50)	−0.425	0.671
	52w	0.50 (0, 11.50)	0 (0, 29.50)	−0.427	0.669
AUC13.9	Baseline	0.30 (0.03, 1.58)	0.30 (0.15, 0.45)	−0.23	0.818
	52w	0 (0, 0.18)	0 (0, 0.65)	−0.513	0.608
MBG	Baseline	11.93 ± 3.00	11.26 ± 2.08	0.598	0.556
	52w	8.63 ± 1.52	8.83 ± 2.00	−0.285	0.778
SDBG	Baseline	2.75 ± 0.65	2.67 ± 0.60	0.317	0.754
	52w	2.24 ± 0.95	2.91 ± 1.31	−1.486	0.151
MAX	Baseline	17.70 ± 2.92	17.18 ± 2.31	0.46	0.65
	52w	14.43 ± 3.47	15.14 ± 3.90	-0.472	0.641
MIN	Baseline	7.19 ± 2.70	6.43 ± 2.46	0.697	0.493
	52w	5.06 ± 1.21	4.46 ± 1.62	1.065	0.298
	Change	−2.30 (−3.08, −0.15)	−2.60 (−4.15, 1.25)	−0.17	0.865
LAGE	Baseline	10.52 ± 1.85	10.74 ± 1.85	−0.29	0.775
	52w	8.98 ± 3.76	10.69 ± 3.86	−1.078	0.292
MAGE	Baseline	6.74 ± 1.99	6.88 ± 2.20	−0.157	0.877
	52w	5.86 ± 2.81	7.09 ± 3.21	−0.997	0.329
*M*-Value	Baseline	26.76 (16.31, 58.85)	24.80 (16.04, 34.97)	−0.481	0.63
	52w	5.19 (2.47, 14.96)	10.75 (4.42, 33.65)	−1.585	0.113
MODD	Baseline	2.33 ± 1.33	2.33 ± 0.83	0.002	0.999
	52w	1.91 ± 0.90	2.19 ± 0.62	−0.82	0.424

PT: Percentage time of blood glucose; AUC: area under the curve; MBG, mean blood glucose; SDBG: standard deviation of blood glucose; MAX: the maximum blood glucose; MIN: the minimum blood glucose; LAGE: the largest amplitude of glycemic excursion; MAGE: mean amplitude of glycemic excursion; MODD: absolute means of daily difference.

**Table 4:** Oxidative stress and inflammatory profile.

Variable		Dulaglutide group (*n* = 16)	Glargine group (*n* = 9)
TNF-a (pg/ml)	Baseline	7.23 (5.92, 8.74)	5.82 (4.89, 7.02)
	52w	5.1 (4.24,7.88)^*∗*^	5.96 ± 1.35
IL-6 (pg/ml)	Baseline	0.87 ± 0.46	0.95 (0.51, 1.72)
	52w	0.64 ± 0.39	0.61 (0.51, 1.08)
PGF-2a (pg/ml)	Baseline	12.28 (10.71, 26.54)	11.05 (7.34, 17.97)
	52w	9.24 (3.62, 19.46)^*∗*^	5.60 (3.39, 9.09)^*∗*^

^∗*p*<0.05^ vs. baseline.

**Table 5:** Microvascular complications and other treatments.

Variable	Baseline				Week 52		
	Dulaglutide (*n* = 16)	Glargine (*n* = 9)	*p*	Dulaglutide (*n* = 16)	Glargine (*n* = 9)	*p*
Diabetic nephropathy, *n* (%)	3	2	0.835	3	3	0.412
Other comorbidities, *n* (%)						
Hyperlipidemia	5	4	0.509	6	4	0.734
Hypertension	11	5	0.509	11	5	0.509
Other treatments, *n* (%)						
CCB	4	3	0.656	4	3	0.656
ACEI/ARB	5	2	0.629	5	2	0.629
Beta-blockers	2	0	0.260	2	0	0.260
Statins	3	2	0.835	4	2	0.876

CCB: calcium channel blockers; ACEI/ARB: angiotensin-converting enzyme inhibitor and/or angiotensin II receptor blocker.

## Data Availability

The data are restricted to researchers approved by ethical committee of Nanjing Hospital affiliated to Nanjing Medical University, Nanjing. Other researcher may access the data upon request and approval of ethical committee of Nanjing Hospital affiliated to Nanjing Medical University, Nanjing.
